# The immune system as a driver of mitochondrial disease pathogenesis: a review of evidence

**DOI:** 10.1186/s13023-022-02495-3

**Published:** 2022-09-02

**Authors:** Allison Hanaford, Simon C. Johnson

**Affiliations:** 1 Center for Integrative Brain Research, Seattle Children’s Research Institute, 1900 9th Ave., JMB-925, Seattle, WA 98101 USA; 2 Department of Laboratory Medicine and Pathology, University of Washington, Seattle, WA USA; 3 Department of Anesthesiology and Pain Medicine, University of Washington, Seattle, WA USA; 4 Department of Neurology, University of Washington, Seattle, WA USA

**Keywords:** Mitochondrial disease, Leigh syndrome, MELAS, Immunity, Genetic disease

## Abstract

**Background:**

Genetic mitochondrial diseases represent a significant challenge to human health. These diseases are extraordinarily heterogeneous in clinical presentation and genetic origin, and often involve multi-system disease with severe progressive symptoms. Mitochondrial diseases represent the most common cause of inherited metabolic disorders and one of the most common causes of inherited neurologic diseases, yet no proven therapeutic strategies yet exist. The basic cell and molecular mechanisms underlying the pathogenesis of mitochondrial diseases have not been resolved, hampering efforts to develop therapeutic agents.

**Main body:**

In recent pre-clinical work, we have shown that pharmacologic agents targeting the immune system can prevent disease in the *Ndufs4*(KO) model of Leigh syndrome, indicating that the immune system plays a causal role in the pathogenesis of at least this form of mitochondrial disease. Intriguingly, a number of case reports have indicated that immune-targeting therapeutics may be beneficial in the setting of genetic mitochondrial disease. Here, we summarize clinical and pre-clinical evidence suggesting a key role for the immune system in mediating the pathogenesis of at least some forms of genetic mitochondrial disease.

**Conclusions:**

Significant clinical and pre-clinical evidence indicates a key role for the immune system as a significant in the pathogenesis of at least some forms of genetic mitochondrial disease.

## Background

Genetic mitochondrial diseases affect over 1 in 4000 individuals, are the most common cause of inherited metabolic disorders, and are a major cause of genetic neurological diseases [1]. Mitochondrial disease can involve a strikingly diverse array of clinical features ranging from primarily single organ involvement, as in Leber’s hereditary optic neuropathy (LHON), to severe multi-system disorders, such as in Leigh syndrome (LS) or mitochondrial encephalomyopathy with lactic acidosis and stroke-like episodes (MELAS) [2–7].

Mitochondrial diseases are characterized by both genetic and clinical heterogeneity. Over 350 disease causing genes have been causally linked to genetic mitochondrial disease, including genes in both the mitochondrial and nuclear genomes [8–12]. The often exceedingly rare individual genetic causes cluster into clinically defined syndromes based on symptoms. Accordingly, there is a great deal of genetic heterogeneity within many individual mitochondrial diseases; for example, over 75 genes have been shown to cause LS alone [13–15]. In general, no clear mechanisms distinguish genes *within* a given clinical syndrome from those in other syndromes. Significant clinical heterogeneity in disease onset, course, and severity is perhaps unsurprising given the complex genetic architecture of mitochondrial diseases, but genetic differences alone have yet to explain the variant clinical progression of individual cases.

Mechanistic explanations for the complex pathogenesis of individual genetic mitochondrial diseases, and for the differential pathogenesis of unique syndromes arising from similar primary mitochondrial defects, have been elusive, and clinically proven therapeutics are lacking. Decades of research focused on the primary molecular sequelae of genetic defects in mitochondrial components, i.e. reduced electron transport chain capacity or ATP production or increased reactive oxygen species production, have yielded a great deal of novel insight into the inner workings of mitochondria, but have failed to explain the pathobiology of genetic mitochondrial disease syndromes, and have provided no meaningful interventions. Pre-clinical studies in mouse, invertebrate, and cell models have also identified various putative approaches to modulating disease in a non-clinical setting including gene therapy, hypoxia, mTOR inhibition (see below), and NAD redox manipulation, but each is limited by unclear translational potential and/or lack of generalizability (in the case of gene therapy, where each of the > 350 genes would require ad hoc approaches) (see [16–19]). While rescuing primary genetic defects in pre-clinical models has become possible in recent years, the lack of *clinical* therapeutic options is in no small part a consequence of the fact that the mechanistic pathobiology of mitochondrial disease has been poorly studied, leaving few options for therapeutic targeting. Identification of common pathogenic processes downstream of the primary genetic defects could provide treatments for genetically distinct forms of mitochondrial disease.

In a recently published study, we demonstrated that immune cells causally drive CNS lesions in the murine model of LS, the most common form of pediatric mitochondrial disease [20]. LS is a particularly severe form of mitochondrial disease, with symptoms including cerebellar ataxia, hypotonia, respiratory dysfunction, lactic acidosis, seizures, and progressive symmetric necrotizing lesions in the brain stem and cerebellum, which are a defining feature of the disease. In this study, we demonstrate that targeting immune cells through high-dose leukocyte-specific inhibitors rescued both central nervous system degeneration and systemic disease. Taken with prior studies probing the benefits of mTOR inhibitors in mitochondrial disease, these data provide strong evidence that the pathogenesis of LS is immune mediated.

The potential importance of these findings for the treatment and understanding of mitochondrial disease is clear, and assessment of current knowledge regarding the relationship between immune function and mitochondrial disease in human patients is prudent. Given the ad-hoc management of mitochondrial disease patients, often as a result of symptom management during extended workups leading to eventual diagnosis, there are a number of case-reports of mitochondrial disease patients being treated with various immune-suppressive interventions. Here, we provide a brief review of these reports, as well as the pre-clinical evidence from mice.

## Main text

### Immune dysfunction in genetic mitochondrial disease

Mitochondrial diseases are remarkably heterogeneous. Clinically distinct mitochondrial disease syndromes such as Leigh syndrome and MELAS differ from each other age of onset, organ system involvement, and specific disease sequelae, but disease course and individual symptoms can differ greatly even among patients with the same diagnosed disorder or causal mutation. Multi-system mitochondrial disorders are most defined by neurologic, metabolic, and muscular symptoms, but mitochondrial defects can impact every organ system, including the immune system.

Altered immune system function is a well-documented possible sequalae of genetic mitochondrial dysfunction, but available data indicate that the nature of immune system involvement is extremely complex and very poorly understood. Recent data, detailed below, clearly demonstrate that aberrant immune activation plays a key causal role in the pathogenesis of some forms of genetic mitochondrial disease (discussed below). However, reduced or impaired immune function is a source of acute medical distress in mitochondrial disease, and infections are a major cause of morbidity and mortality in these patients; many experience recurrent infections and delayed recovery following infection, and in some studies sepsis and pneumonia are the most common proximal causes of death [21–24]. A retrospective study of about 100 ‘definite’ or ‘probable’ mitochondrial disease patients (patients with diagnosis supported by biochemical or genetic studies) at Massachusetts General Hospital provides an overview of the range and severity of immune symptoms experienced by these patients: ~ 40% experienced serious or recurring infections, with over 10% suffering from one or more episodes of sepsis or systemic inflammatory response syndrome (SIRS), while, at the same time, ~ 40% of these patients experienced atopy and/or were diagnosed with an autoimmune disease [22].

Many mitochondrial disease patients are evaluated for immune deficiency during the diagnosis process. In a recent study, 56% of patients were found to be seronegative for antibodies to two or more vaccine preventable diseases, despite more than 80% adherence to the recommended vaccination schedule, indicating that mitochondrial disease patients either fail to develop immunity following vaccination or are unable to maintain immunity [23]. These striking data not only raise clinically and scientifically significant questions about the nature of immune system defects in mitochondrial disease, but also highlight how much we do not yet know about these disorders.

In addition to reports associating mitochondrial disease in toto with immune dysfunction, some individual mitochondrial diseases are clearly associated with specific immune defects. Low white blood cell counts (leukopenia) have been reported in multiple forms of mitochondrial disease. Neutropenia (a type of leukopenia) is reported to impact 90% of patients with Barth syndrome, for example, which is caused by defects in genes involved in production of the mitochondria-specific structural lipid cardiolipin [25, 26]. Specific deficiencies in CD8 + T-cell and NK cell, which are related to recurrent infections, have been reported in a patient with mitochondrial DNA depletion [27]. In line with these data, MELAS patients show a depletion of pathogenic heteroplasmic mutation load in T-cells, consistent with a ‘purifying’ selection against mutant mtDNA a result of their altered function [28]. Cytochrome C oxidase (COX) activity has been shown to influence T-cell functions in a sub-population dependent manner and lead to immune deficiency, while COX defects in mice impair B-cell positive selection [29, 30]. Also in mice, mitochondrial CIII function has been shown to be important for antigen-specific T-cell activation and invariant NK cell development and function [31, 32]. Our aim here is to summarize evidence linking immune activity to disease pathogenesis in genetic mitochondrial disease, so a full review of immunometabolism and the role of mitochondria in immune system function is beyond our scope; many high-quality literature reviews expand on these topics (for some examples, see [33–36]).

### Evidence for immune involvement in the pathogenesis of mitochondrial disease

#### Clinical evidence—signs of immune involvement in non-immune sequela of disease

**Fig. 1:**
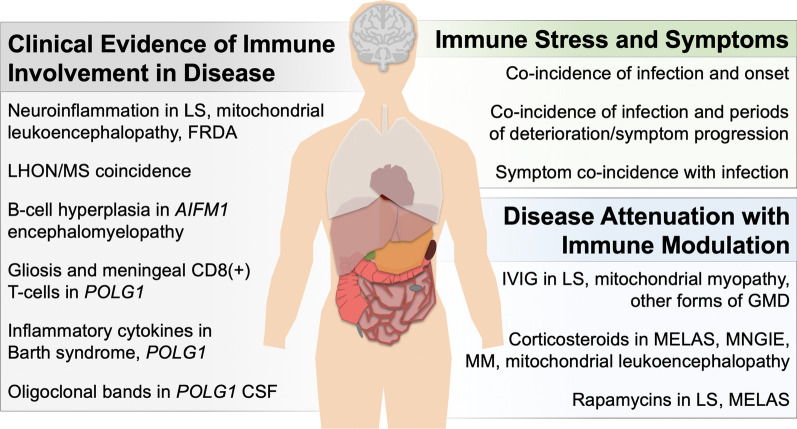
Clinical evidence of immune-mediated disease pathogenesis in the setting of genetic mitochondrial disease

An array of evidence from case reports and natural history studies suggests immune involvement in the pathogenesis of genetic mitochondrial diseases (see Fig. 1, Table 1).

**Table 1:** Clinical studies reporting immune targeting interventions in mitochondrial diseases

Disorder/syndrome	Immunomodulatory treatment	Reported outcome	Citation
Mitochondrial myopathy	IVIG	Significant clinical improvement	[71, 81, 82, 85, 86]
	Methylprednisolone	Significant clinical improvement	[81]
	Prednisone	Substantial improvement sustained after steroid cessation	[82]
	Dexamethasone, prednisone	Improvement in exercise tolerance and muscle strength	[85]
	Prednisone	Significant clinical recovery; symptom return with dose reduction	[86]
Mitochondrial leukoencephalopathy	Methylpredisolone	Significant clinical improvement	[56]
ND4-related demyelinating syndrome	Plasmapheresis, steroids, IVIG	Improvement with plasmapheresis, but not steroids or IVIG	[72]
mtDNA depletion syndrome	IVIG + steroids	Stabilization of disease	[72]
DARS2-related demyelinating syndrome	Rituximab + steroids	Stablization. Symptoms returned after cessation of treatment	[72]
ATP6A-related Leigh syndrome	Plasmapheresis followed by regular IVIG	Substantial clinical improvement, symptoms returned when plasmapheresis ceased. Improvement upon treatment resumption and with maintenance on regular IVIG	[73]
MELAS	Dexamethasone	Sustained clinical improvement. Relapse upon cessation of steroids	[76]
	Prednisolone	Sustained clinical improvement, relapse upon dose reduction/cessation	[77]
	Dexamethasone and prednisone	Sustained clinical improvement, relapse upon dose reduction/cessation	[78]
	Corticosteroids	Sustained clinical improvement	[79]
	Prednisone	Significant clinical improvement	[75]
	Everolimus	No response	[87]
		Improvement (patients post-kidney transplant)	[88]
Mitochondrial encephalomyopathy	Prednisolone	Significant clinical improvement. Relapse when dose decreased	[83]
Mitochondrial myopathy with eosinophilia	Corticosteroids	Improvement. Symptoms relapsed when steroids ceased, improvement with subsequent treatment	[84]
NDUFS4-related Leigh syndrome	Everolimus	Sustained improvement	[87]

In LHON, progressive damage and functional compromise of the optic nerve results in vision loss. Vascular abnormalities and swelling can be detected early in disease. Though direct evidence for immune involvement in humans is sparse, some data suggests that early-stage LHON is responsive to corticosteroids [37, 38]. LHON often presents in patients also diagnosed with MS, with the dual diagnosis of MS and LHON known as Harding’s syndrome or LHON-MS [39]. MS is an autoimmune disease which is not fully understood, likely has multiple distinct causes, and is not considered a genetic mitochondrial disease; however, in MS occurs much more often with LHON than would occur by chance in Harding’s syndrome, and in this situation the MS symptoms can be putatively causally linked to the mitochondrial defect driving the LHON pathology [39, 40]. MS has been causally linked to inflammation, while evidence increasingly supports a role for mitochondrial dysfunction [41–43]. Harding’s syndrome provides an example of a disease clearly linked to both mitochondrial defects and inflammation.

Mitochondrial disease arising from defects in mitochondrial apoptosis inducing factor 1 *(AIFM1)* can cause a wide range of symptoms including encephalomyopathy, cerebellar ataxia, peripheral neuropathy, etc. [44–51]. Clinical evidence for immune involvement is minimal (see pre-clinical section below), but a patient with particularly severe disease presented with follicular bronchiolitis, a rare non-neoplastic B-cell hyperplasia typically associated with genetic immune defects, acquired immunodeficiencies, or autoimmune disease [44, 52].

In Barth syndrome, while patients experience recurrent bacterial infections due to neutropenia, they also have persistently elevated plasma levels of the inflammatory cytokine interleukin 6 (IL-6), consistent with chronic inflammation, which are thought to contribute to muscle-wasting [53–55].

Mitochondrial leukoencephalopathy are mitochondrial disorders with CNS white matter involvement. MRI of the brains of patients with these disorders reveal white matter pathology including contrast enhancement and diffusion restriction consistent with blood brain barrier breakdown and neuroinflammation [56]. Reactive microgliosis was also observed by histology in a subset of patients, though there was no evidence of peripheral immune cells in the CSF.

Evidence for chronic inflammation has also been reported in patients with mutations in *POLG1* (encoding a subunit of the mtDNA polymerase): two case reports found oligoclonal banding in CSF versus plasma consistent with CSF autoimmunity and typically seen in multiple sclerosis, where a causal role for immune involvement is well accepted [57–61]. Elevated CSF levels of the inflammatory cytokines interferon gamma (IFNγ), IL-6, and IL-8 have also been observed in *POLG1* disease [62]. Brain biopsies of *POLG1* patients have provided direct evidence of gliosis and perivascular CD8( +) T-cells in the meninges [63].

Defects in thymidine kinase 2 (TK2) also impact mtDNA, leading to a mtDNA depletion syndrome with progressive myopathy. Transcriptomic analysis of muscle biopsies from TK2 deficiency patients has revealed increased expression of genes associated with inflammation [64].

Inflammation appears to play a role in the pathogenesis of the mitochondria-associated iron accumulation disease Friedreich ataxia (FRDA). Microgliosis has been reported in the dorsal root ganglia of FRDA patients, a known site of FRDA neuropathology [65]. Microglial activation has also been reported in FRDA patients in brain regions associated with neuropathology using PET scanning for ^18^F-FEMPA, a high-affinity ligand for the microglia-specific 18-kDa Translocator protein (TPSO) [66, 67]. ^18^F-FEMPA signal intensity correlates with age of disease onset, supporting a causal role for neuroinflammation in FRDA symptoms [66]. FRDA patients also show elevated plasma IL-6, and FRDA patient blood transcriptomic profiles show upregulated innate immune responses [66, 68].

#### Clinical evidence—immune targeting interventions

In addition to its use in treating immunoglobulin deficiencies, intravenous immunoglobulin (IVIG) therapy is a well-documented immune-suppressing therapeutic strategy used in autoimmune disorders such as systemic lupus erythematosus, antiphospholipid syndrome, Kawasaki disease, demyelinating diseases, autoimmune neuromuscular disease, and scleroderma [69, 70]. In mitochondrial disease, a patient with a confirmed mitochondrial myopathy was found to have muscle T-cell infiltration upon biopsy and showed significant clinical improvement after IVIG therapy [71]. In another case report, three children with genetically and clinically distinct forms of mitochondrial disease were treated with immune targeting therapies. One responded well to corticosteroids alone, another stabilized with corticosteroids and the B-cell depleting drug rituximab, another failed to respond to corticosteroids but showed marked improvement following IVIG [72]. Perhaps most dramatically, an adult-onset Leigh syndrome patient was given plasmapheresis to treat a suspected autoimmune disease and experienced resolution of symptoms prior to the confirmation of a Leigh syndrome diagnosis, with a known causal homoplasmic ATP6A mtDNA variant and characteristic CNS lesions [73]. Strikingly, the patient remitted following therapy, but with IVIG once again showed substantial improvements in symptoms. The authors concluded simply that the mechanism of benefit was unknown, but that ATP6A Leigh syndrome may involve underlying autoimmune mechanisms.

Corticosteroids, as mentioned above, also appear to provide significant, and at times persistent, benefits in genetically and clinically distinct forms of mitochondrial disease [74]. These include MELAS [75–79], mitochondrial neurogastrointestinal encephalopathy (MNGIE) [80], mitochondrial myopathy [81], mitochondrial encephalomyopathy [82, 83], mitochondrial leukoencephalopathy [56], and other forms of mitochondrial disease [84–86]. The clinical reversal of disease was so striking in some cases, as in the full reversal of CNS symptoms in MELAS with subsequent dependency on corticosteroids to prevent relapse, that authors suggested corticosteroids should be standard treatments in mitochondrial disease [76]. However, while corticosteroids may benefit many forms of mitochondrial disease, a review of case reports found that they are likely detrimental in one form of mitochondrial disease, Kearns-Sayre syndrome [74]. Whether a different immune targeting approach would yield benefits, or immune involvement is not universal in mitochondrial disease, remains to be determined.

In response to preclinical data from the *Ndufs4*(KO) mouse (detailed below), mechanistic target of rapamycin (mTOR) inhibitors have also recently been tested in small cohorts of mitochondrial disease patients. Rapamycins (rapamycin and analogs such as sirolimus, everolimus, and formulations such as ABI-009/nab-rapamycin) have immune suppressive actions mechanistically distinct from corticosteroids and calcineurin inhibitors, and are FDA approved for use in preventing transplant rejection and graft versus host disease (Drug Approval Package: Rapamune (Sirolimus), Application No.: 021083). A recent trial reported the use of everolimus in two pediatric mitochondrial disease patients reported mixed responses [87]. A 2 year-old Leigh syndrome patient homozygous for the known pathogenic c.355G > C (pAsp119His) mutation in *NDUFS4* responded strikingly well to mTOR inhibition therapy, with a reversal of brain lesions and striking recovery of gross motor function which persisted through ~ 20 months of therapy. The other patient, a 69 month-old MELAS patient with high heteroplasmy levels of the common m.3243A > G variant, did not appear to benefit. In a separate study four post-transplant MELAS patients in terminal decline were transitioned from calcineurin inhibitors to mTOR inhibitors, and all four showed substantial improvement in over the following months [88].

#### Immunologic stress as a possible catalyst for symptoms

In a survey of high priority questions among mitochondrial disease patients and caregivers in the UK, two of the top 10 questions were ‘what factors could trigger the start of mitochondrial disease in people who have a genetic mutation?’ and ‘why are people with the same genetic mutation affected so differently in mitochondrial disease?’ [89]. While the answer to these questions is complex, immune functions appear to be one potent contributor to both onset and severity of genetic mitochondrial disease.

Mitochondrial disease can present prenatally or at birth, but in many patients, symptoms do not appear until later. Adult-onset mitochondrial disease is well-documented, even in classically pediatric syndromes such as LS. Even in those with pediatric onset it is notable that symptoms are frequently absent at birth in many forms of mitochondrial disease. Disease onset can vary widely both when comparing different clinically defined mitochondrial disease syndromes, as with the generally adult-onset MELAS versus typically pediatric onset Leigh syndrome, but also occur between patients with the same clinical disorder. It is hard to reconcile any post-natal onset of serious multi-system degenerative disorders such as LS, particularly in cases of adult-onset disease, with models where mitochondrial bioenergetics or ROS directly drive disease pathobiology.

While a robust link has not yet been established, viral infection and fever have been reported to coincide with symptom onset in mitochondrial disease [21, 90–95]. For example, in three unrelated patients with *POLG* mutations, symptom onset followed infection with human herpesvirus 6 or *Borrelia* [96, 97]. These patients all presented with severe seizures and rapid progressive neurodegeneration despite antivirals and antibiotics treatment and were eventually diagnosed with mitochondrial disease caused by *POLG*. *Borrelia* infection has also been associated with the onset of disease in a case of LHON [98]. A study of 14 pediatric patients with mitochondrial leukoencephalopathy in India described febrile illness as the inciting event in 57% of patients [56]. It has been hypothesized that this link is due to the energetic stress associated with induction of an immune response [91]. In light of the evidence for an immunologic origin of disease in LS it seems reasonable that it is the upregulated immune responses themselves that trigger disease onset. Testing this possibility will require careful study using animal models.

Similarly, while genetic mitochondrial diseases are progressive, the progression of symptoms is not linear—patients experience periods of relative stability interrupted by periods of deterioration. Reasons for ebbs and flow in disease are likely complex, but infections are one important factor. In a study of 130 LS patients in Europe, 61% of acute exacerbations resulting in hospitalization were found to result from infections [99]. In another small study it was found that infection appeared a few days prior to neurodegenerative events in a striking ~ 70% of cases [21]. Moreover, there was a temporal delay of about a week between infection and neurodegenerative event—a timeframe the authors note is similar to that observed in Reye syndrome and suggest could be related to the induction of inflammatory cytokines or cellular mediators of immunity. These findings are notable in light of recent findings linking mitochondrial function to T-cells immune regulatory function in mice (discussed below).

Finally, it appears possible that some mitochondrial defects only present when uncovered by an immunologic insult. In at least one case, mitochondrial leukoencephalopathy which may have been precipitated by infection appeared reversible after recovery from acute illness—an infant with a *DARS2* mutation experienced dramatic neurological deterioration after a respiratory tract infection at 9 months of age but gradually improved several months after resolution of the infection until nearly complete recovery by 14 months [100].

Together, these findings support a link between immune activity and symptom onset and progression in genetic mitochondrial disease. Though the precise mechanisms underpinning this link are yet to be defined, and may differ by form of mitochondrial disease or immune stress, some clues exist. For example, there is a substantial body of literature on sepsis demonstrating that the septic state can cause mitochondrial dysfunction, and that sepsis-induced mitochondrial dysfunction mediates some of the pathologic consequences of sepsis including lactic acidosis and multiple organ failure (see [101–105] for detailed reviews on this topic). Energetics defects, ROS, and metabolic decompensation are thought to be the major pathways involved in this setting. In addition, some cytokines, including interferon, IL1β, CXCL1, MCP1, IL6, and IL4, have been shown to directly influence mitochondria and drive mitochondrial dysfunction in mice or cultured cells [106–108]. Accordingly, multiple, non-mutually exclusive, processes are likely involved in linking immune activation to symptom onset or worsening in mitochondrial disease.

#### Pre-clinical studies

**Fig. 2:**
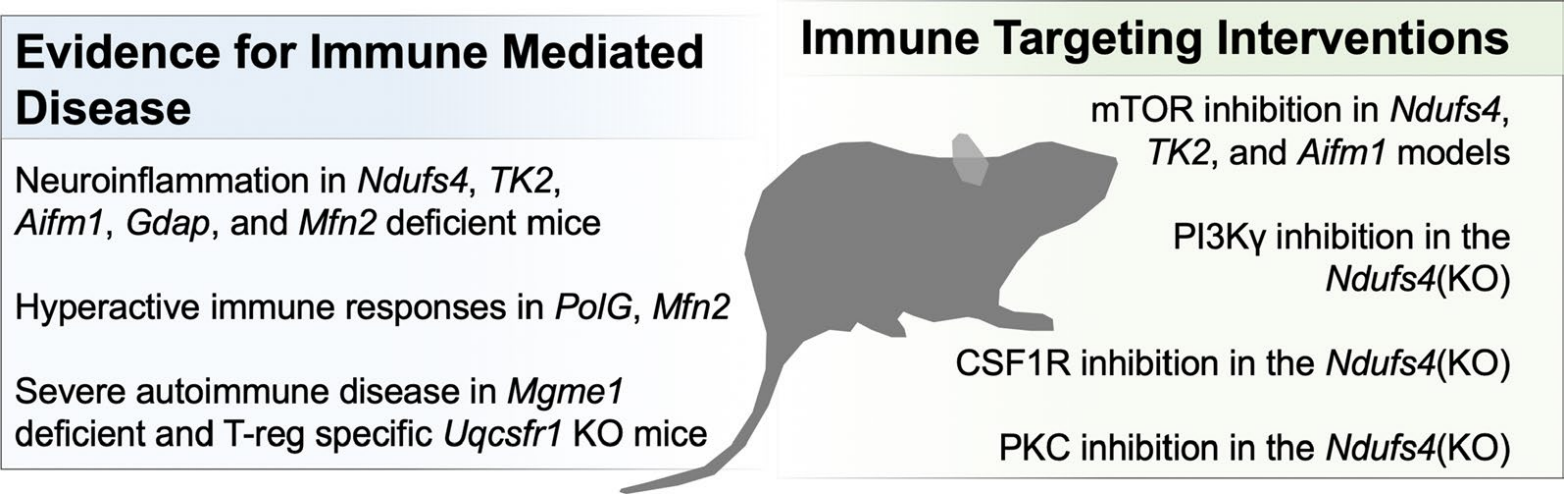
Evidence for immune involvement in murine models of genetic mitochondrial disease

Murine models of mitochondrial disease also strongly support an immune-centric model for the pathogenesis of genetic mitochondrial disease (Fig. 2):

In 2013 and 2015, we published studies demonstrating that high-dose oral or injected rapamycin significantly delays disease onset and extends survival in the *Ndufs4*(KO) mouse model of LS [109–111]. The benefits of mTOR inhibition were found to be independent of mitochondrial function—mitochondrial respiratory capacity and ETC CI assembly, stability, and levels were all unaffected by treatment—but the precise mechanism underlying the benefits of rapamycin were unresolved at the time.

In subsequent efforts aimed at defining the role of mTOR in LS, we tested upstream signaling through PI3K. Class I phosphoinositide 3-kinases (PI3K) mediate signaling from cell-membrane bound receptors to AKT/mTOR and exist in forms defined by their catalytic (p110) subunit: PI3Kα, PI3Kβ, PI3Kδ, and PI3Kγ. Of these, only inhibition of PI3Kγ significantly was found to attenuate disease, with an effect size identical to that provided by rapamycin [112]. Critically, PI3Kγ is an immune-cell specific factor, PI3Kγ deficiencies lead to immune deficiency in mice and humans, and PI3Kγ inhibitors have been used in a variety of autoimmune models including lupus and multiple sclerosis [113, 114].

In light of the PI3Kγ results, *Ndufs4*(KO) mice were treated with the CSF1R inhibitor pexidartinib/PLX3397. CSF1R is involved in hematopoietic cell differentiation into monocytes/macrophages and survival of macrophages, including tissue macrophages and microglia, and is highly expressed by microglia in inflammatory conditions [115]. CSF1R inhibition rescued disease, including both a complete prevention of CNS lesions and a rescue of peripheral symptoms such as metabolic dysfunction and cachexia [112]. Very recently, the partial benefits provided by low doses of PLX3397 have been independently reproduced by another group [116]. Together, these data provide strong evidence that LS is an immune-mediated disease.

In light of these data, various other studies support a model where genetic mitochondrial diseases are driven in part by immune-mediated processes. In a phospho-proteomic study aimed at probing the role of mTOR in LS, phosphorylation of the pro-inflammatory protein kinase C (PKC) and activation of pro-inflammatory PKC regulated targets, including the NF-κB pathway, was reduced by rapamycin treatment in the *Ndufs4*(KO). PKC inhibitors were found to slightly, but significantly, attenuate disease and extend survival in this model, supporting a causal role for inflammation [117].

Whether the immune system contributes to the pathogenesis of other forms of primary genetic mitochondrial disease remains to be causally assessed, but available data does seem to support this possibility.

Alopecia, aberrant bone resorption, and hepatic metabolic dysfunction are peripheral symptoms of disease in the *Ndufs4*(KO) that have been shown to be driven by immune cell hyperactivation and macrophage dysfunction [118].

Treatment of TK2 deficient mouse model of mtDNA depletion with rapamycin, for example, significantly increased survival, as was seen in the *Ndufs4*(KO) [119]. Similarly, the polymerase gamma (PolG) deficient ‘mutator’ mouse model of mtDNA instability has been shown to have aberrantly hyperactive innate immune responses, with type-I interferon responses contributing at significantly to the phenotype [120]. In a more recent third model of mtDNA instability, loss of the mitochondrial genome maintenance exonuclease *Mgme1*, which causes adult-onset mitochondrial disease in humans, results in severe autoimmune disease with prominent inflammatory renal disease [121]. Infiltrating T- and B-cells were present in these renal lesions, and elevated levels of circulating blood IL-6, TNFα, IL-10, and IL-2 indicating systemic inflammation.

In the *Aifm1* deficient Harlequin mouse model of mitochondrial disease, mice develop overt microgliosis with elevated levels of the proinflammatory cytokines TNFα and IL-1β in brain regions impacted by disease, reminiscent of *Ndufs4*(KO) cortical neuropathology but without the characteristic symmetric lesions [112, 122, 123]. Importantly, mTOR activity was found to be hyperactive, and rapamycin rescued grip strength and metabolic defects, supporting a role for mTOR in mitochondrial disease beyond *Ndufs4*(KO) [122]. Similarly, evidence gathered using the *Ndufs4* deficiency as a model of optic neuropathy resulting from ETC CI dysfunction revealed that loss of retinal ganglion cells is at least partially driven by inflammation and responsive to mTOR inhibition [124].

Charcot-Marie-Tooth (CMT) disease is the most common inherited neuropathy. While the disease is both clinically and genetically diverse, some cases of CMT have been causally linked to genes encoding mitochondria proteins, including mitofusion 2 (*MFN2)* and ganglioside-induced differentiation-associated protein 1 (*GDAP1*) [125]. Mice lacking *Gdap*(-/-) develop astrocytosis and microgliosis in the spinal cord, with increased expression of pro-inflammatory *TNF-α* and *Cxcl10* [126]. Mice heterozygous for a disease-causing *Mfn2* mutation also present with microgliosis in the optic nerves and in the lumbar spinal cord [127]. When immunologically challenged with LPS, these mice have a greater increase in serum TNFα and IL-6, and an exacerbated immune response in the lumbar spinal cord and optic nerve after LPS challenge, compared to control animals [127].

#### Pre-clinical evidence that immunologic stress is a catalyst for symptoms

Few animal studies have tested the relationship between immune activation and symptoms in mitochondrial disease, though available data clearly supports a causal link. One important recent study aimed at directly assessing the bioenergetic costs of infection demonstrated that viral infection leads to metabolic decompensation and mitochondrial hepatopathy in hepatocyte specific *Cox10* knockout mice [128]. Moreover, metabolic dysfunction could be induced in cultured *Cox10* knockout hepatocytes by TNFα at concentrations with no impact on control cells, while virus-induced hepatotoxicity in vivo was ameliorated by the TNFα receptor inhibitor etanercept. These data demonstrate one mechanism for immune-mediated mitochondrial disease symptom onset precipitated by infection. It remains to be seen whether TNFα plays a prominent role in other sequelae of genetic mitochondrial dysfunction, and if additional cytokines are involved.

### Mitochondria and immune regulation in mice

While these findings have highlighted the importance of immune cell actions in the pathogenesis of primary genetic mitochondrial disorders, evidence from basic research in immune cell regulation by mitochondrial metabolism has made this link ever clearer. For example, a naturally occurring mouse mtDNA variant in *mt-Atp8* has been found to control susceptibility to disease in two different models of autoimmune skin disease [129]. Perhaps most striking, cell-specific deletion of the mitochondrial disease associated ETC CIII component encoding *Uqcsrf1* restricted to regulatory T-cells has been found to blunt regulatory T-cell suppressive function to such a severe degree that mice die from rampant autoimmunity within weeks of birth [130]. Notably, regulatory T-cells were present at normal levels, demonstrating that mitochondrial dysfunction in these cells led to severe defects in function without simply causing their depletion.

### Mitochondrial origins and sources of autoreactivity

Mitochondria are highly immunogenic through several distinct pathways, including potent innate immune pathways. Mitochondria arose during eukaryotic evolution as an endosymbiotic intracellular organelle with bacterial origins. This bacterial origin has resulted in eukaryotic organisms relying on mitochondria as a critically important intracellular organelle which, somewhat remarkably, happens to have retained multiple components that are sensed as foreign if aberrantly released.

Mitochondrial DNA, RNA, and proteins can all be recognized as foreign, pathogenic, material. Sensing pathways include detection of mitochondria-derived nucleotides, including mtDNA, by TLR9, which recognizes unmethylated CpG dinucleotides found in bacterial DNA; sensing of mitochondrial encoded RNA transcripts by TLR’s, evolved to sense viral or bacterial RNA; sensing of mtDNA by the intracellular cGAS-STING-IRF3-dependent pathway; and disruption of the mitochondrial outer membrane associated mitochondrial antiviral signaling protein, MAVS, which mediates extra- and intra- cellular pathogen RNA sensing through complex, mitochondrial ROS and morphology related, events [131–133].

Mitochondrial proteins can also activate innate immune pathways. Formylated methionine is an amino acid present in pathogens and mitochondria, and extra-mitochondrial formylated peptides can induce innate immune responses through formyl-peptide receptors (FPRs). While protective against pathogens, FPR signaling mediates tissue inflammation and pathology in settings of ischemia–reperfusion, celiac disease, and pulmonary chemical insults, and have been shown to contribute to neurodegenerative disease [134, 135].

Worthy of a separate lengthy discussion (see reviews [136–139]), these pathways provide ample rationale for further study. Their roles, if any, in genetic mitochondrial disease remain largely unexplored.

## Outstanding questions and future considerations

A role for the immune system in causally contributing to the pathogenesis of at least a subset of genetic mitochondrial diseases appears increasingly likely as various roles for mitochondria in immune function and autoimmunity are resolved. However, a number of critical questions remain unanswered, and immune-based interventions, particularly those which are untargeted in nature, carry serious caveats and limitations.

In particular, the clinical studies and data discussed here are predominately case reports, which are considered weak evidence for clinical practice [140]. Case report data must also be interpreted carefully in light of the fact that fluctuations in lesions (by MRI) and clinical status have been documented in Leigh syndrome in the absence of any intervention [99, 141, 142]. While there are significant barriers to performing well-controlled clinical trials in the setting of ultra-rare genetic diseases, these challenges should not mask the limitations of case reports of observational studies.

None of the discussed pre-clinical therapeutics or clinical immune-targeting therapeutics are approved for use in treating genetic mitochondrial disease. As non-clinicians, we make no effort to advocate for any of the described treatments. Rather, our point in this review is to provide an assessment of current evidence suggesting that immune-mediated processes play a causal role in the pathogenesis of mitochondrial disease. Significant further work is needed to elucidate the precise mechanisms linking mitochondrial dysfunction, immune dysregulation, and pathology. Efforts to understand the basic biology of mitochondrial disease may lead to more targeted intervention strategies, while future trials may determine that approaches such as plasmapheresis, a minimally invasive and well-tolerated non-FDA regulated procedure, may prove beneficial in some acute settings.

## Conclusions

In the context of mitochondrial disease, the critical role of mitochondria as modulators of immunity has been a remarkably underappreciated role of these organelles, with the focus on bioenergetics and ROS dominating attempts at therapeutic intervention. A 2012 review concluded that there was no evidence to support the use of *any* vitamin or cofactor in treating mitochondrial disease [143]. Ten years later, solid evidence supporting these approaches is still elusive. Given the failure of antioxidants, nutritional supplements, and pharmacologic approaches to increasing energetic output to meaningfully alter disease course in either animal models or human clinical trials, it is clear that new approaches should be explored [144]. The data reviewed here strongly suggest that the role of the immune system in mitochondrial disease warrants substantially greater attention.

While we have focused our discussion here on genetic mitochondrial diseases, the findings in this field are likely to be relevant to other forms of disease where mitochondrial dysfunction plays a causal role. In addition to primary genetic disorders, mitochondrial dysfunction is involved in pathology in the setting of environmental exposures, complex multigenic diseases/traits, and age-related disease [145, 146]. Environmental exposures to known mitochondrial toxins, such as the mitochondrial complex I toxins rotenone and annonacin, are associated Parkinson’s disease, Alzheimer’s disease, and progressive supranuclear palsy [147, 148]. Complex multigenic traits include diseases where many genetic loci influence disease, but through individually weak effects often best demonstrate by genome-wide association studies [145]. Age-related disease clearly linked to both mitochondrial function and immune activity include Parkinson’s, Alzheimer’s, and Multiple Sclerosis, which was briefly mentioned above. While beyond our scope here, we suspect that defining the interplay between immune system activity and mitochondrial function in these diseases will be critical to understanding their pathogenesis. Studies in genetic mitochondrial disease is likely to yield important insight relevant to each of these settings.

## Data Availability

Not applicable.

## Abbreviations

CMT: Charcot-Marie-tooth
COX: Cytochrome c oxidase
CSF: Cerebral spinal fluid
FRDA: Friedreich’s ataxia
LHON: Leber’s hereditary optic neuropathy
LS: Leigh syndrome
MELAS: Mitochondrial encephalomyopathy with lactic acidosis and stroke-like episodes
MNGIE: Mitochondrial neurogastrointestinal encephalopathy
mtDNA: Mitochondrial DNA
PET: Positron emission tomography
SIRS: Systemic inflammatory response syndrome

## References

[1] Gorman GS (2016). Mitochondrial diseases. Nat Rev Dis Primers.

[2] El-Hattab AW, Adesina AM, Jones J, Scaglia F (2015). MELAS syndrome: clinical manifestations, pathogenesis, and treatment options. Mol Genet Metab.

[3] Hilo W, Jabaly-Habib H, Modi N, Briscoe D (2013). Leber’s hereditary optic neuropathy. Harefuah.

[4] Yu-Wai-Man P, Chinnery PF. Leber Hereditary Optic Neuropathy. 2000 Oct 26 [Updated 2021 Mar 11]. In: Adam MP, Everman DB, Mirzaa GM, et al., editors. GeneReviews® [Internet]. Seattle (WA): University of Washington, Seattle; 1993-2022. Available from: https://www.ncbi.nlm.nih.gov/books/NBK1174/. 20301353

[5] Thorburn DR, Rahman J, Rahman S. Mitochondrial DNA-Associated Leigh Syndrome and NARP. 2003 Oct 30 [Updated 2017 Sep 28]. In: Adam MP, Everman DB, Mirzaa GM, et al., editors. GeneReviews® [Internet]. Seattle (WA): University of Washington, Seattle; 1993-2022. Available from: https://www.ncbi.nlm.nih.gov/books/NBK1173/20301352

[6] Rahman S, Thorburn D. In: Adam MP et al., editors. GeneReviews(R). Seattle, 1993.

[7] Chinnery PF. Primary Mitochondrial Disorders Overview. 2000 Jun 8 [Updated 2021 Jul 29]. In: Adam MP, Everman DB, Mirzaa GM, et al., editors. GeneReviews® [Internet]. Seattle (WA): University of Washington, Seattle; 1993-2022. Available from: https://www.ncbi.nlm.nih.gov/books/NBK1224/.

[8] Alston CL, Rocha MC, Lax NZ, Turnbull DM, Taylor RW (2017). The genetics and pathology of mitochondrial disease. J Pathol.

[9] Chinnery PF. Primary Mitochondrial Disorders Overview. 2000 Jun 8 [Updated 2021 Jul 29]. In: Adam MP, Everman DB, Mirzaa GM, et al., editors. GeneReviews® [Internet]. Seattle (WA): University of Washington, Seattle; 1993-2022. Available from: https://www.ncbi.nlm.nih.gov/books/NBK1224/.

[10] Viscomi C, Bottani E, Zeviani M (2015). Emerging concepts in the therapy of mitochondrial disease. Biochim Biophys Acta.

[11] Frazier AE, Thorburn DR, Compton AG (2019). Mitochondrial energy generation disorders: genes, mechanisms, and clues to pathology. J Biol Chem.

[12] Ng YS (2021). Mitochondrial disease in adults: recent advances and future promise. Lancet Neurol.

[13] Ruhoy IS, Saneto RP (2014). The genetics of Leigh syndrome and its implications for clinical practice and risk management. Appl Clin Genet.

[14] Gerards M, Sallevelt SC, Smeets HJ (2016). Leigh syndrome: resolving the clinical and genetic heterogeneity paves the way for treatment options. Mol Genet Metab.

[15] Lake NJ, Compton AG, Rahman S, Thorburn DR (2016). Leigh syndrome: one disorder, more than 75 monogenic causes. Ann Neurol.

[16] van de Wal MAE (2022). Ndufs4 knockout mouse models of Leigh syndrome: pathophysiology and intervention. Brain.

[17] Reynaud-Dulaurier R (2020). Gene replacement therapy provides benefit in an adult mouse model of Leigh syndrome. Brain.

[18] Silva-Pinheiro P, Cerutti R, Luna-Sanchez M, Zeviani M, Viscomi C (2020). A single intravenous injection of AAV-PHP.B-hNDUFS4 ameliorates the phenotype of Ndufs4 (-/-) mice. Mol Ther Methods Clin Dev.

[19] Jain IH (2016). Hypoxia as a therapy for mitochondrial disease. Science.

[20] Stokes JC, Bornstein RL, James K, Park KY, Spencer KA, Vo K, Snell JC, Johnson BM, Morgan PG, Sedensky MM, Baertsch NA, Johnson SC. Leukocytes mediate disease pathogenesis in the Ndufs4(KO) mouse model of Leigh syndrome. JCI Insight. 2022;7(5):e156522. 10.1172/jci.insight.156522. PMID: 35050903; PMCID: PMC8983133. 10.1172/jci.insight.156522 PMC8983133 35050903

[21] Edmonds JL (2002). The otolaryngological manifestations of mitochondrial disease and the risk of neurodegeneration with infection. Arch Otolaryngol Head Neck Surg.

[22] Walker MA (2014). Predisposition to infection and SIRS in mitochondrial disorders: 8 years’ experience in an academic center. J Allergy Clin Immunol Pract.

[23] Kruk SK (2019). Vulnerability of pediatric patients with mitochondrial disease to vaccine-preventable diseases. J Allergy Clin Immunol Pract.

[24] Eom S (2017). Cause of death in children with mitochondrial diseases. Pediatr Neurol.

[25] Finsterer J (2007). Hematological manifestations of primary mitochondrial disorders. Acta Haematol.

[26] Clarke SL (2013). Barth syndrome. Orphanet J Rare Dis.

[27] Reichenbach J (2006). Fatal neonatal-onset mitochondrial respiratory chain disease with T cell immunodeficiency. Pediatr Res.

[28] Walker MA (2020). Purifying selection against pathogenic mitochondrial DNA in human T cells. N Engl J Med.

[29] Tarasenko TN (2017). Cytochrome c oxidase activity is a metabolic checkpoint that regulates cell fate decisions during T cell activation and differentiation. Cell Metab.

[30] Chen D (2021). Coupled analysis of transcriptome and BCR mutations reveals role of OXPHOS in affinity maturation. Nat Immunol.

[31] Weng X, Kumar A, Cao L, He Y, Morgun E, Visvabharathy L, Zhao J, Sena LA, Weinberg SE, Chandel NS, Wang CR. Mitochondrial metabolism is essential for invariant natural killer T cell development and function. Proc Natl Acad Sci U S A. 2021;118(13):e2021385118. 10.1073/pnas.2021385118. PMID: 33753493; PMCID: PMC8020658. 10.1073/pnas.2021385118 PMC8020658 33753493

[32] Sena LA (2013). Mitochondria are required for antigen-specific T cell activation through reactive oxygen species signaling. Immunity.

[33] Kapnick SM, Pacheco SE, McGuire PJ (2018). The emerging role of immune dysfunction in mitochondrial diseases as a paradigm for understanding immunometabolism. Metabolism.

[34] Joseph AM, Monticelli LA, Sonnenberg GF (2018). Metabolic regulation of innate and adaptive lymphocyte effector responses. Immunol Rev.

[35] Makowski L, Chaib M, Rathmell JC (2020). Immunometabolism: from basic mechanisms to translation. Immunol Rev.

[36] Van den Bossche J, O'Neill LA, Menon D (2017). Macrophage immunometabolism: Where are we (going)?. Trends Immunol.

[37] Lee C (2020). Leber's hereditary optic neuropathy following unilateral painful optic neuritis: a case report. BMC Ophthalmol.

[38] Mauri E (2018). Subclinical leber’s hereditary optic neuropathy with pediatric acute spinal cord onset: more than meets the eye. BMC Neurol.

[39] Parry-Jones AR, Mitchell JD, Gunarwardena WJ, Shaunak S (2008). Leber’s hereditary optic neuropathy associated with multiple sclerosis: harding’s syndrome. Pract Neurol.

[40] Pfeffer G, Burke A, Yu-Wai-Man P, Compston DA, Chinnery PF (2013). Clinical features of MS associated with leber hereditary optic neuropathy mtDNA mutations. Neurology.

[41] Barcelos IP, Troxell RM, Graves JS (2019). Mitochondrial dysfunction and multiple sclerosis. Biology (Basel).

[42] Gonzalez LF, Bevilacqua LE, Naves R (2021). Nanotechnology-based drug delivery strategies to repair the mitochondrial function in neuroinflammatory and neurodegenerative diseases. Pharmaceutics.

[43] Picone P, Nuzzo D (2022). Promising treatment for multiple sclerosis: mitochondrial transplantation. Int J Mol Sci.

[44] Morton SU (2017). AIFM1 mutation presenting with fatal encephalomyopathy and mitochondrial disease in an infant. Cold Spring Harb Mol Case Stud.

[45] Bogdanova-Mihaylova P (2019). Clinical spectrum of AIFM1-associated disease in an Irish family, from mild neuropathy to severe cerebellar ataxia with colour blindness. J Peripher Nerv Syst.

[46] Peng Q (2022). Case report: a novel intronic mutation in AIFM1 associated with fatal encephalomyopathy and mitochondrial disease in infant. Front Pediatr.

[47] Hu B (2017). A novel missense mutation in AIFM1 results in axonal polyneuropathy and misassembly of OXPHOS complexes. Eur J Neurol.

[48] Elrharchi S (2020). Novel mutation in AIFM1 gene associated with X-linked deafness in a Moroccan family. Hum Hered.

[49] Edgerley K (2021). AIFM1-associated X-linked spondylometaphyseal dysplasia with cerebral hypomyelination. Am J Med Genet A.

[50] Diodato D (2016). A novel AIFM1 mutation expands the phenotype to an infantile motor neuron disease. Eur J Hum Genet.

[51] Ardissone A (2015). A slowly progressive mitochondrial encephalomyopathy widens the spectrum of AIFM1 disorders. Neurology.

[52] Tashtoush B, Okafor NC, Ramirez JF, Smolley L (2015). Follicular bronchiolitis: a literature review. J Clin Diagn Res.

[53] Wilson LD, Al-Majid S, Rakovski CS, Schwindt CD (2012). Higher IL-6 and IL6:IGF ratio in patients with barth syndrome. J Inflamm (London).

[54] Payette H (2003). Insulin-like growth factor-1 and interleukin 6 predict sarcopenia in very old community-living men and women: the Framingham heart study. J Am Geriatr Soc.

[55] Tanaka T, Narazaki M, Kishimoto T (2014). IL-6 in inflammation, immunity, and disease. Cold Spring Harb Perspect Biol.

[56] Bindu PS (2018). Mitochondrial leukoencephalopathies: A border zone between acquired and inherited white matter disorders in children?. Mult Scler Relat Disord.

[57] Slee M (2011). A novel mitochondrial DNA deletion producing progressive external ophthalmoplegia associated with multiple sclerosis. J Clin Neurosci.

[58] Echaniz-Laguna A (2010). POLG1 variations presenting as multiple sclerosis. Arch Neurol.

[59] Link H, Huang YM (2006). Oligoclonal bands in multiple sclerosis cerebrospinal fluid: an update on methodology and clinical usefulness. J Neuroimmunol.

[60] Deisenhammer F, Zetterberg H, Fitzner B, Zettl UK (2019). The cerebrospinal fluid in multiple sclerosis. Front Immunol.

[61] Degos B (2014). POLG mutations associated with remitting/relapsing neurological events. J Clin Neurosci.

[62] Hasselmann O (2010). Cerebral folate deficiency and CNS inflammatory markers in Alpers disease. Mol Genet Metab.

[63] Nolte KW (2013). Early muscle and brain ultrastructural changes in polymerase gamma 1-related encephalomyopathy. Neuropathology.

[64] Kalko SG (2014). Transcriptomic profiling of TK2 deficient human skeletal muscle suggests a role for the p53 signalling pathway and identifies growth and differentiation factor-15 as a potential novel biomarker for mitochondrial myopathies. BMC Genomics.

[65] Koeppen AH, Ramirez RL, Becker AB, Mazurkiewicz JE (2016). Dorsal root ganglia in Friedreich ataxia: satellite cell proliferation and inflammation. Acta Neuropathol Commun.

[66] Khan W (2022). Neuroinflammation in the cerebellum and brainstem in friedreich ataxia: an [18F]-FEMPA PET study. Mov Disord.

[67] Ghadery C (2017). Microglial activation in Parkinson’s disease using [(18)F]-FEPPA. J Neuroinflamm.

[68] Nachun D (2018). Peripheral blood gene expression reveals an inflammatory transcriptomic signature in Friedreich’s ataxia patients. Hum Mol Genet.

[69] Dalakas MC (2004). The use of intravenous immunoglobulin in the treatment of autoimmune neuromuscular diseases: evidence-based indications and safety profile. Pharmacol Ther.

[70] Shoenfeld Y, Katz U (2005). IVIg therapy in autoimmunity and related disorders: our experience with a large cohort of patients. Autoimmunity.

[71] Mancuso M (2013). An “inflammatory” mitochondrial myopathy. A case report Neuromuscul Disord.

[72] Hacohen Y (2015). Acute evidence of CNS inflammation in patients with mitochondrial diseases. (P.4053). Neurology.

[73] Chuquilin M, Govindarajan R, Peck D, Font-Montgomery E (2016). Response to immunotherapy in a patient with adult onset leigh syndrome and T9176C mtDNA mutation. Mol Genet Metab Rep.

[74] Finsterer J, Frank M (2015). Glucocorticoids for mitochondrial disorders. Singapore Med J.

[75] Walcott BP (2012). Steroid responsive A3243G mutation MELAS: clinical and radiographic evidence for regional hyperperfusion leading to neuronal loss. Neurologist.

[76] Gubbay SS, Hankey GJ, Tan NT, Fry JM (1989). Mitochondrial encephalomyopathy with corticosteroid dependence. Med J Aust.

[77] Hsu CC (1995). CPEO and carnitine deficiency overlapping in MELAS syndrome. Acta Neurol Scand.

[78] Rossi FH, Okun M, Yachnis A, Quisling R, Triggs WJ (2002). Corticosteroid treatment of mitochondrial encephalomyopathies. Neurologist.

[79] Schiariti M, Cacciola MT, Pangallo A, Ciancia F, Puddu PE (2009). Delayed pericarditis and cardiac tamponade associated with active-fixation lead pacemaker in the presence of mitochondrial myopathy and Ockham’s razor. J Cardiovasc Med (Hagerstown).

[80] Bedlack RS (2004). MNGIE neuropathy: five cases mimicking chronic inflammatory demyelinating polyneuropathy. Muscle Nerve.

[81] Heiman-Patterson TD (1997). Biochemical and genetic studies in a family with mitochondrial myopathy. Muscle Nerve.

[82] Mastaglia FL, Thompson PL, Papadimitriou JM (1980). Mitochondrial myopathy with cardiomyopathy, lactic acidosis and response to prednisone and thiamine. Aust N Z J Med.

[83] Fox C, Dunne J (1993). Corticosteroid responsive mitochondrial encephalomyopathy. Aust N Z J Med.

[84] Finsterer J, Hoger F (2009). Multi-system mitochondrial disorder with recurrent steroid-responsive eosinophilia. Rheumatol Int.

[85] Shapira Y, Cederbaum SD, Cancilla PA, Nielsen D, Lippe BM (1975). Familial poliodystrophy, mitochondrial myopathy, and lactate acidemia. Neurology.

[86] Skoglund RR (1979). Reversible alexia, mitochondrial myopathy, and lactic acidemia. Neurology.

[87] Sage-Schwaede A (2019). Exploring mTOR inhibition as treatment for mitochondrial disease. Ann Clin Transl Neurol.

[88] Johnson SC (2019). mTOR inhibitors may benefit kidney transplant recipients with mitochondrial diseases. Kidney Int.

[89] Thomas RH (2022). Research priorities for mitochondrial disorders: current landscape and patient and professional views. J Inherit Metab Dis.

[90] Lee YJ, Hwang SK, Kwon S (2019). Acute necrotizing encephalopathy in children: a long way to go. J Korean Med Sci.

[91] Niyazov DM, Kahler SG, Frye RE (2016). Primary mitochondrial disease and secondary mitochondrial dysfunction: importance of distinction for diagnosis and treatment. Mol Syndromol.

[92] Porta F (2021). SLC25A19 deficiency and bilateral striatal necrosis with polyneuropathy: a new case and review of the literature. J Pediatr Endocrinol Metab.

[93] Wang HS, Huang SC (2001). Acute necrotizing encephalopathy of childhood. Chang Gung Med J.

[94] Wei Y, Wang L (2018). Adult-onset Leigh syndrome with central fever and peripheral neuropathy due to mitochondrial 9176T>C mutation. Neurol Sci.

[95] Wu TS (1993). Leigh disease (subacute necrotizing encephalomyelopathy): report of one case. Zhonghua Min Guo Xiao Er Ke Yi Xue Hui Za Zhi.

[96] Al-Zubeidi D (2014). Fatal human herpesvirus 6-associated encephalitis in two boys with underlying POLG mitochondrial disorders. Pediatr Neurol.

[97] Gaudo P (2020). Infectious stress triggers a POLG-related mitochondrial disease. Neurogenetics.

[98] Macarez R (2005). Onset of Leber’s hereditary optic neuropathy in association with borreliosis. J Fr Ophtalmol.

[99] Sofou K (2014). A multicenter study on leigh syndrome: disease course and predictors of survival. Orphanet J Rare Dis.

[100] Kohler C (2015). Early-onset leukoencephalopathy due to a homozygous missense mutation in the DARS2 gene. Mol Cell Probes.

[101] Yang H, Zhang Z (2021). Sepsis-induced myocardial dysfunction: the role of mitochondrial dysfunction. Inflamm Res.

[102] Sun J (2019). Mitochondria in sepsis-induced AKI. J Am Soc Nephrol.

[103] Kohoutova M, Dejmek J, Tuma Z, Kuncova J (2018). Variability of mitochondrial respiration in relation to sepsis-induced multiple organ dysfunction. Physiol Res.

[104] Gu M, Mei XL, Zhao YN (2021). Sepsis and cerebral dysfunction: BBB damage, neuroinflammation, oxidative stress, apoptosis and autophagy as key mediators and the potential therapeutic approaches. Neurotox Res.

[105] Levy RJ (2007). Mitochondrial dysfunction, bioenergetic impairment, and metabolic down-regulation in sepsis. Shock.

[106] Qualls AE, Southern WM, Call JA (2021). Mitochondria-cytokine crosstalk following skeletal muscle injury and disuse: a mini-review. Am J Physiol Cell Physiol.

[107] Pattnaik B (2016). IL-4 promotes asymmetric dimethylarginine accumulation, oxo-nitrative stress, and hypoxic response-induced mitochondrial loss in airway epithelial cells. J Allergy Clin Immunol.

[108] Shan B, Vazquez E, Lewis JA (1990). Interferon selectively inhibits the expression of mitochondrial genes: a novel pathway for interferon-mediated responses. EMBO J.

[109] Johnson SC (2013). mTOR inhibition alleviates mitochondrial disease in a mouse model of leigh syndrome. Science.

[110] Johnson SC (2014). Translational medicine. A target for pharmacological intervention in an untreatable human disease. Science.

[111] Johnson SC (2015). Dose-dependent effects of mTOR inhibition on weight and mitochondrial disease in mice. Front Genet.

[112] Stokes JC (2022). Leukocytes mediate disease pathogenesis in the Ndufs4(KO) mouse model of Leigh syndrome. JCI Insight.

[113] Lanahan SM, Wymann MP, Lucas CL (2022). The role of PI3Kgamma in the immune system: new insights and translational implications. Nat Rev Immunol.

[114] Thian M (2020). Germline biallelic PIK3CG mutations in a multifaceted immunodeficiency with immune dysregulation. Haematologica.

[115] Han J (2022). Inhibition of colony stimulating factor-1 receptor (CSF-1R) as a potential therapeutic strategy for neurodegenerative diseases: opportunities and challenges. Cell Mol Life Sci.

[116] Aguilar K, Comes G, Canal C, Quintana A, Sanz E, Hidalgo J. Microglial response promotes neurodegeneration in the Ndufs4 KO mouse model of Leigh syndrome. Glia. 2022. 10.1002/glia.24234. Epub ahead of print. PMID: 35770802. 10.1002/glia.24234 PMC9544686 35770802

[117] Martin-Perez M (2020). PKC downregulation upon rapamycin treatment attenuates mitochondrial disease. Nat Metab.

[118] Jin Z, Wei W, Yang M, Du Y, Wan Y (2014). Mitochondrial complex I activity suppresses inflammation and enhances bone resorption by shifting macrophage-osteoclast polarization. Cell Metab.

[119] Siegmund SE (2017). Low-dose rapamycin extends lifespan in a mouse model of mtDNA depletion syndrome. Hum Mol Genet.

[120] Lei Y (2021). Elevated type I interferon responses potentiate metabolic dysfunction, inflammation, and accelerated aging in mtDNA mutator mice. Sci Adv.

[121] Milenkovic D (2022). Mice lacking the mitochondrial exonuclease MGME1 develop inflammatory kidney disease with glomerular dysfunction. PLoS Genet.

[122] Wischhof L (2018). A disease-associated Aifm1 variant induces severe myopathy in knockin mice. Mol Metab.

[123] Fernandez-de la Torre M (2020). Exercise training and neurodegeneration in mitochondrial disorders: insights from the harlequin mouse. Front Physiol.

[124] Yu AK (2015). Mitochondrial complex I deficiency leads to inflammation and retinal ganglion cell death in the Ndufs4 mouse. Hum Mol Genet.

[125] Klein CJ (2020). Charcot-Marie-tooth disease and other hereditary neuropathies. Continuum (Minneap Minn).

[126] Fernandez-Lizarbe S (2019). Neuroinflammation in the pathogenesis of axonal Charcot-Marie-tooth disease caused by lack of GDAP1. Exp Neurol.

[127] Stavropoulos F (2021). Aberrant mitochondrial dynamics and exacerbated response to neuroinflammation in a novel mouse model of CMT2A. Int J Mol Sci.

[128] Jestin M (2020). Mitochondrial disease disrupts hepatic allostasis and lowers the threshold for immune-mediated liver toxicity. Mol Metab.

[129] Schilf P (2021). A mitochondrial polymorphism alters immune cell metabolism and protects mice from skin inflammation. Int J Mol Sci.

[130] Weinberg SE (2019). Mitochondrial complex III is essential for suppressive function of regulatory T cells. Nature.

[131] West AP (2015). Mitochondrial DNA stress primes the antiviral innate immune response. Nature.

[132] Kruger A (2015). Human TLR8 senses UR/URR motifs in bacterial and mitochondrial RNA. EMBO Rep.

[133] Onomoto K, Onoguchi K, Yoneyama M (2021). Regulation of RIG-I-like receptor-mediated signaling: interaction between host and viral factors. Cell Mol Immunol.

[134] Vacchelli E, Le Naour J, Kroemer G (2020). The ambiguous role of FPR1 in immunity and inflammation. Oncoimmunology.

[135] Busch L, Vieten S, Brodel S, Endres K, Bufe B (2022). Emerging contributions of formyl peptide receptors to neurodegenerative diseases. Biol Chem.

[136] Mills EL, Kelly B, O'Neill LAJ (2017). Mitochondria are the powerhouses of immunity. Nat Immunol.

[137] Angajala A (2018). Diverse roles of mitochondria in immune responses: novel insights into immuno-metabolism. Front Immunol.

[138] Breda CNS, Davanzo GG, Basso PJ, Saraiva Câmara NO, Moraes-Vieira PMM (2019). Mitochondria as central hub of the immune system. Redox Biol.

[139] Weinberg SE, Sena LA, Chandel NS (2015). Mitochondria in the regulation of innate and adaptive immunity. Immunity.

[140] Kamel C, McGahan L, Mierzwinski-Urban M, Embil J. Preoperative Skin Antiseptic Preparations and Application Techniques for Preventing Surgical Site Infections: A Systematic Review of the Clinical Evidence and Guidelines [Internet]. Ottawa (ON): Canadian Agency for Drugs and Technologies in Health; 2011. PMID: 24354038. 24354038

[141] Arii J, Tanabe Y (2000). Leigh syndrome: serial MR imaging and clinical follow-up. AJNR Am J Neuroradiol.

[142] Alves C (2020). Pediatric leigh syndrome: neuroimaging features and genetic correlations. Ann Neurol.

[143] Pfeffer G, Majamaa K, Turnbull DM, Thorburn D, Chinnery PF. Treatment for mitochondrial disorders. Cochrane Database Syst Rev. 2012;2012(4):CD004426. 10.1002/14651858.CD004426.pub3. PMID: 22513923; PMCID: PMC7201312. 10.1002/14651858.CD004426.pub3 PMC7201312 22513923

[144] Almannai M, El-Hattab AW, Ali M, Soler-Alfonso C, Scaglia F (2020). Clinical trials in mitochondrial disorders, an update. Mol Genet Metab.

[145] Johnson SC (2017). Network analysis of mitonuclear GWAS reveals functional networks and tissue expression profiles of disease-associated genes. Hum Genet.

[146] Bornstein R, Gonzalez B, Johnson SC (2020). Mitochondrial pathways in human health and aging. Mitochondrion.

[147] Lannuzel A, Ruberg M, Michel PP (2008). Atypical Parkinsonism in the Caribbean island of Guadeloupe: etiological role of the mitochondrial complex I inhibitor annonacin. Mov Disord.

[148] Radad K (2019). Rotenone: from modelling to implication in Parkinson’s disease. Folia Neuropathol.

